# Heart Failure: Diagnosis, Severity Estimation and Prediction of Adverse Events Through Machine Learning Techniques

**DOI:** 10.1016/j.csbj.2016.11.001

**Published:** 2016-11-17

**Authors:** Evanthia E. Tripoliti, Theofilos G. Papadopoulos, Georgia S. Karanasiou, Katerina K. Naka, Dimitrios I. Fotiadis

**Affiliations:** a Department of Biomedical Research, Institute of Molecular Biology and Biotechnology, FORTH, GR 45110 Ioannina, Greece; b Unit of Medical Technology and Intelligent Information Systems, University of Ioannina, GR 45110 Ioannina, Greece; c Michaelidion Cardiac Center, University of Ioannina, GR 45110 Ioannina, Greece; d 2nd Department of Cardiology, University of Ioannina, GR 45110 Ioannina, Greece

**Keywords:** Heart failure, Diagnosis, Prediction, Severity estimation, Classification, Data mining

## Abstract

Heart failure is a serious condition with high prevalence (about 2% in the adult population in developed countries, and more than 8% in patients older than 75 years). About 3–5% of hospital admissions are linked with heart failure incidents. Heart failure is the first cause of admission by healthcare professionals in their clinical practice. The costs are very high, reaching up to 2% of the total health costs in the developed countries. Building an effective disease management strategy requires analysis of large amount of data, early detection of the disease, assessment of the severity and early prediction of adverse events. This will inhibit the progression of the disease, will improve the quality of life of the patients and will reduce the associated medical costs. Toward this direction machine learning techniques have been employed. The aim of this paper is to present the state-of-the-art of the machine learning methodologies applied for the assessment of heart failure. More specifically, models predicting the presence, estimating the subtype, assessing the severity of heart failure and predicting the presence of adverse events, such as destabilizations, re-hospitalizations, and mortality are presented. According to the authors' knowledge, it is the first time that such a comprehensive review, focusing on all aspects of the management of heart failure, is presented.

## 1 Introduction

Heart failure (HF) is a complex clinical syndrome and not a disease. It prevents the heart from fulfilling the circulatory demands of the body, since it impairs the ability of the ventricle to fill or eject blood. It is characterized by symptoms, such as breathlessness, ankle swelling and fatigue that may be accompanied by signs, for example elevated jugular venous pressure, pulmonary crackles, and peripheral edema, caused by structural and/or functional cardiac or non-cardiac abnormalities. HF is a serious condition associated with high morbidity and mortality rates. According to the European Society of Cardiology (ESC), 26 million adults globally are diagnosed with HF, while 3.6 million are newly diagnosed every year. 17–45% of the patients suffering from HF die within the first year and the remaining die within 5 years. The related to HF management costs are approximately 1–2% of all healthcare expenditure, with most of them linked with recurrent hospital admissions [1], [2], [3].

The increased prevalence, the escalated healthcare costs, the repeated hospitalizations, the reduced quality of life (QoL) and the early mortality have transformed HF to an epidemic in Europe and worldwide and highlight the need for early diagnosis (detection of the presence of HF and estimation of its severity) and effective treatment. In clinical practice, medical diagnosis, including carefully history and physical examination, is supported by ancillary tests, such as blood tests, chest radiography, electrocardiography and echocardiography [4]. The combination of data produced by the above procedure of diagnosis resulted in the formulation of several criteria (*e.g.* Framingham, Boston, the Gothenburg and the ESC criteria) determining the presence of HF [5]. Once the diagnosis of HF is established, the experts classify the severity of HF using either the New York Heart Association (NYHA) or the American College of Cardiology/American Heart Association (ACC/AHA) Guidelines classification systems, since this classification allows them to determine the most appropriate treatment (medication treatment, guidelines regarding nutrition and physical activity exercising) to be followed [6].

Although there is a significant progress in understanding the complex pathophysiology of HF, the quantity and complexity of data and information to be analyzed and managed convert the accurate and efficient diagnosis of HF and the assessment of therapeutic regimens to quite challenging and complicated tasks. Those factors, in combination with the positive effects of early diagnosis of HF (which allows experts to design an effective and possibly successful treatment plan, prevents condition worsening, affects positively the patient's health, improves patient's QoL and contributes to decrease of medical costs) are the reasons behind the enormous increase of the application of machine learning techniques to analyze, predict and classify medical data. Classification methods are among the data mining techniques that have gained the interest of research groups. Accurate classification of disease stage or etiology or subtypes allows treatments and interventions to be delivered in an efficient and targeted way and permits assessment of the patient's progress.

**Fig. 1:**
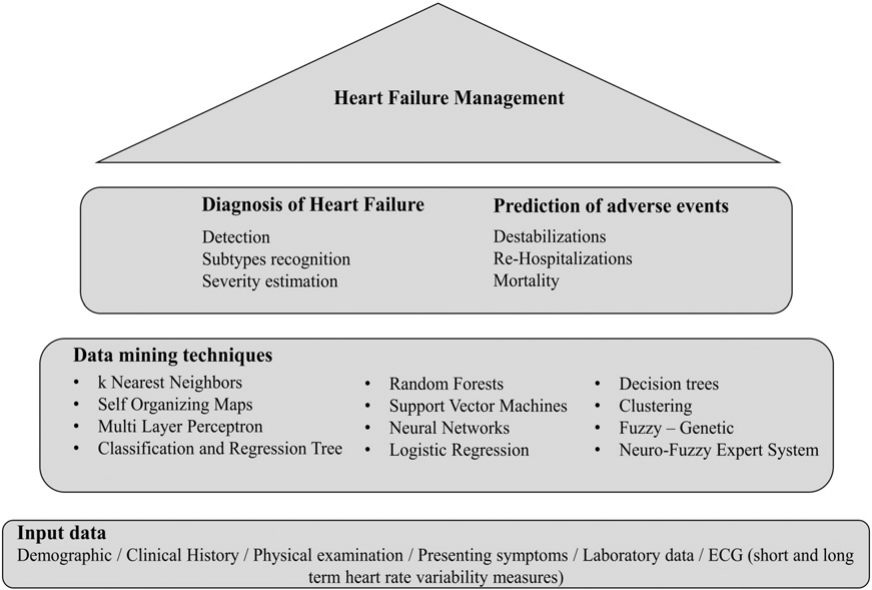
Overview of studies on heart failure management.

Focusing on HF, different data mining techniques have been employed to differentiate the patients with HF from controls, to recognize the different HF subtypes (*e.g.* HF with reduced ejection fraction, HF with preserved ejection fraction) and to estimate the severity of HF (NYHA class) (Fig. 1). Additionally, data mining techniques can be advantageous even if HF is being diagnosed at a late stage, where the therapeutic benefits of interventions and the prospect of survival are limited, since they allow the timely prediction of mortality, morbidity and risk of readmission. Data recorded in the subjects' health record, expressing demographic information, clinical history information, presenting symptoms, physical examination results, laboratory data, electrocardiogram (ECG) analysis results, are employed. An extended review of the studies reported in the literature addressing the above mentioned issues (HF detection, severity estimation, prediction of adverse events) through the utilization of machine learning techniques is presented in this paper.

The systematic literature review was based on sources like i) PubMeD, ii) Scopus, iii) ScienceDirect, iv) Google Scholar, v) Web of Science (WoS) using as keywords the phrases “detection of HF”, “severity estimation of HF”, “HF subtypes classification”, “prediction of HF destabilizations”, “prediction of HF relapses”, “prediction of HF mortality”, “prediction of HF re-hospitalizations”.

The studies reported in the literature were selected based on the following criteria: i) focus on heart failure and no any other heart disease, ii) are written in English language, iii) are published from 2000 (inclusive) until present, iv) cover different geographical locations, v) are employing machine learning techniques, vi) employ Electronic Health Records, published databases, observational, trial, *etc.* for the development and validation, vii) provide information regarding the evaluation measures and the validation method that was followed and, viii) the response feature is either differentiation of subjects to normal and HF or differentiation of subjects to different HF subtypes or estimation of the severity of HF or estimation of the destabilization or estimation of re-admission or estimation of mortality. There is no restriction regarding the time frame of the prediction. Furthermore, studies addressing both aspects of HF management (*e.g.* detection and severity estimation of HF) were also included in this review. Studies not fulfilling more than one of the above mentioned criteria were excluded.

## 2 Detection of HF

According to the ESC guidelines [1], the algorithm to diagnose HF in a non-acute setting is the following. First the probability of HF based on prior clinical history of the patient, the presenting symptoms, physical examination, and resting ECG is estimated. If all elements are normal, HF is highly unlikely. If at least one element is abnormal, plasma Natriuretic Peptides should be measured. This measurement allows the experts to identify those patients who need echocardiography. The process of diagnosis of HF can be: (i) less time consuming, (ii) supported and (iii) performed with the same accuracy by the applications of machine learning techniques on the available data. More specifically, the detection of HF is expressed as a two class classification problem where the output of the classifiers is the presence or not of HF.

Most of the studies reported in the literature focus on the utilization of heart rate variability (HRV) that is a measure to classify a subject as normal or as patient with HF. Those methods are presented in Table 1. The main difference between those methods is related to the HRV features which are employed to detect HF.

**Fig. 2:**
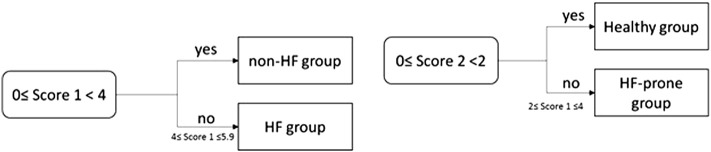
Flow chart of the score model proposed by Yang et al. 2010 [14].

Yang et al. 2010 [19] proposed a scoring model which allows the detection of HF and the assessment of its severity. More specifically, two Support Vector Machines (SVM) models were built. The first model detects the presence or not of HF (Non-HF group *vs.* HF group). In case the subject belongs to the non- HF group, the second model classifies the patients to a Healthy group or to a HF-prone group. The output of the SVM models was mapped to a score value (it is described in Section 4 since the study focuses in the severity estimation of HF). If the score value, produced by mapping the output of the first model (Score 1), is lower than 4 (score interval: 0–4), then the subject belongs to the non-HF group. If Score 1 is > 4 (score interval: 4–5.9), then the subject has HF (HF group). If the Score 1 is lower than 4 and the Score 2 (score produced by mapping the output of the second SVM model) is lower than 2 (score interval: 0–2), then the patient belongs to the Healthy group. If Score 1 is lower than 4 and the Score 2 is > 2 (score interval: 2–4), then the subject belongs to HF-prone group (Fig. 2).

Gharehchopogh et al. 2011 [20] utilized neural networks (NN) and a set of 40 subjects in order to detect HF. For each subject, gender, age, blood pressure, smoking habit and its annotation as normal or patient were available. 38 out of 40 subjects were correctly classified resulting thus to True Positive Rate 95.00%, False Positive Rate 9.00%, Precision 95.00%, Recall 95.00%, F-measure 94.00% and Area Under Curve (AUC) 95%.

Son et al. 2012 [4] studied the discrimination power of 72 variables in differentiating congestive heart failure (CHF) patients from those with dyspnea, and the risk factor Pro Brain Natriuretic Peptides (Pro-BNP). Rough sets and logistic regression were employed for the reduction of the feature space. Then a decision tree based classification was applied to the produced by the previous step feature set. The experimental results showed that the rough sets based decision-making model had accuracy 97.5%, sensitivity 97.2%, specificity 97.7%, positive predictive value 97.2%, negative predictive value 97.7%, and area under ROC curve 97.5%, while the corresponding values for the logistic regression decision-making model were accuracy 88.7%, sensitivity 90.1%, specificity 87.5%, positive predictive value 85.3%, negative predictive value 91.7%, and area under receiver operating characteristic (ROC) curve 88.8%.

Masetic et al. 2016 [21] applied Random Forests algorithm to long-term ECG time series in order to detect CHF. ECG signals were acquired from the Beth Israel Deaconess Medical Center (BIDMC) Congestive Heart Failure and the PTB Diagnostic ECG databases, both freely available on PhysioNet [22], while normal heartbeats were taken from 13 subjects from MIT–BIH Arrhythmia database.^1^ Features were extracted from ECG using the autoregressive Burg method. Besides Random Forests, the authors evaluated, on the same dataset, C4.5, SVM, Artificial Neural Networks (ANN) and *k*-Nearest Neighbors (*k-*NN) classifiers and the performance of the classifiers in terms of sensitivity, specificity, accuracy, F-measure and ROC curve were recorded and compared. The authors have chosen Random Forests due to its very good accuracy in classifying a subject as normal or CHF.

Wu et al. 2010 [23] and Aljaaf et al. 2015 [2] move one step forward and attempt to predict the presence of HF. Wu et al. 2010 [23] modeled detection of HF more than 6 months before the actual date of clinical diagnosis. In order this to be achieved, data from electronic health records of the Geisinger Clinic were employed. The electronic health records included data representing demographic, health behavior, use of care, clinical diagnosis, clinical measures, laboratory data, and prescription orders for anti-hypertensive information. The information was expressed by 179 independent variables. The authors compared SVM, Boosting, and logistic regression models for their ability to early predict the HF. Before the application of classifiers, feature selection was performed. A different selection procedure was followed depending on the classifier. For logistic regression, variable selection was based on minimizing the Akaike information criterion (AIC) and the Bayesian information criterion (BIC), while the L1-norm variable selection technique was used in the case of SVM. AUC was measured and the results indicated that the AUCs were similar for logistic regression and Boosting. The highest median AUC (77.00%) was observed for logistic regression with BIC and Boosting with less strict cut off.

Aljaaf et al. 2015 [2] proposed a multi-level risk assessment of developing HF. The proposed model could predict five risk levels of HF (1: No risk, 2: Low risk, 3: Moderate risk, 4: High risk, 5: Extremely high risk) using C4.5 decision tree classifier. The Cleveland Clinic Foundation heart disease dataset^2^ was used. The authors enhance the dataset with three new attributes - risk factors, namely obesity, physical activity and smoking. The dataset included 160 instances of risk level 1, 35 instances of risk level 2, 54 instances of risk level 3, 35 instances of risk level 4 and 13 instances of risk level 5. For the evaluation of the C4.5 classifier a 10-fold cross-validation procedure was followed. The overall precision of the proposed approach is 86.30%, while the precision for predicting each one of the above mentioned risk levels is 89.00, 86.50, 72.00, 90.90 and 100.00%, respectively.

Zheng et al. 2015 [24] proposed a computer assisted system for the diagnosis of CHF. The computer assisted system employs Least Squares SVM (LS-SVM) and it is trained and tested utilizing heart sound and cardiac reverse features. The results of the LS-SVM classifier were compared with those produced by ANN and Hidden Markov Models indicating thus the superiority of LS-SVM approach.

A short presentation of the above mentioned studies is provided in Table 2.

## 3 HF Subtypes Classification

Once HF is detected, the etiology or the subtypes of HF can be estimated. According to HF guidelines, the etiology of HF is diverse within and among world regions. There is no agreed single classification system for the causes of HF, with much overlap between potential categories. HF manifests at least two major subtypes, which are commonly distinguished based on the measurement of the left ventricular ejection fraction (LVEF) [25]. Patients with LVEF larger or equal to 50% are characterized as patients with HF with preserved ejection fraction (HFpEF), while patients with LVEF lower than 40% are characterized as patients with HF with reduced ejection fraction (HFrEF). When the LVEF lies between 40 and 49% the patient belongs to so called “gray zone”, which is defined as HF with mid-range ejection fraction (HFmrEF).

Machine learning techniques have been applied to classify HF subtypes. This approach of classification of HF subtypes started the last 3 years. Austin et al. 2013 [26] classified HF patients according to two disease subtypes (HFpEF *vs.* HFrEF) using different classification methods. More specifically, classification trees, bagged classification trees, Random Forests, boosted classification trees and SVM were employed. The training of the classifiers was performed using the EFFECT-1 sample of Enhanced Feedback for Cardiac Treatment (EFFECT) study, while for the validation of the classifiers the EFFECT-2 sample was used. The two samples consist of 9.943 and 8.339 patients hospitalized with a diagnosis of HF, respectively. Removing subjects with missing values and subjects whom ejection fraction could not be determined, 3.697 patients for training and 4.515 patients for testing were finally employed. For each patient, 34 variables were recorded expressing information regarding demographic characteristics, vital signs, presenting signs and symptoms, laboratory data and previous medical history. The results indicate that patients can be classified into one of the two mutually exclusive subtypes with 69.6% positive predictive value using the Random Forests classifier.

Betanzos et al. 2015 [25] applied machine learning techniques to classify HF subtypes using the concept of Volume Regulation Graph (VRG) domain rather than by the single use of ejection fraction (EF). More specifically, they used both the metric EF and the basic variables that define the EF, namely end systolic volume (ESV) and end diastolic volume (EDV). This approach allowed them to overcome the limitations inherent to the use of EF which neglects the importance of left ventricular cavity volume. From those data, the end systolic volume index (ESVI) was computed and through the application of machine learning techniques, the validity of ESVI as an index for discriminating between the HFpEF and the HFrEF patients was examined. Both supervised and unsupervised techniques were applied. K-means using Euclidean distance, Expectation - Maximization (EM) and sequential Information Bottleneck algorithm (sIB) were used to perform discrimination in an unsupervised manner. Supervised classifiers, such as SVM, SVMPEGASOS, Nearest Neighbors (IB1) and NNGE, which is a nearest neighbor-like algorithm using non nested generalized exemplars, rule based algorithm OneR, C4.5, PART, and Naive Bayes classifier, were tested and compared. The authors employed two datasets for the evaluation of the above mentioned machine learning techniques. The first dataset included data from 48 real patients (35 belong to the class HFpEF and 13 to the class HFrEF), while the second dataset includes simulated data, generated using Monte Carlo simulation approach, that correspond to 63 instances (34 from class HFpEF and 29 from class HFrEF). The results of the unsupervised methods revealed interesting dividing patterns of the two subtypes, while the SVM PEGASOS algorithm was opted for the classification of the patients, since it produced the best results in terms of training and test error. Based on those results, Betanzos et al. 2015 [25] concentrated on SVMPEGASOS algorithm toward examining how the results are differentiated when patients belonging to the “gray zone” are included. They set different cutoff points (EF at 40, 45, 50, and 55%). The SVM PEGASOS model was trained using the first dataset described previously and it was tested on a new dataset including simulated data corresponding to 403 instances, among which 150 refer to class HFpEF, 137 refer to class HFrEF and 116 refer to HFmrEF. The utilization of the different cutoff points differentiate the number of samples belonging to the two classes. The results indicated that ESV can act as a discriminator even when patients with HFmrEF are included.

Isler 2016 [27] performed a heart rate variability analysis in order to distinguish patients with systolic CHF from patients with diastolic CHF. More specifically, short-term HRV measures were given as input to nearest neighbors and multi-layer perceptron classifiers. Eight different configurations were applied (No heart rate normalization and no MINMAX normalization, heart rate normalization and no MINMAX normalization, No heart rate normalization and MINMAX normalization, Heart rate normalization and MINMAX normalization). 18 patients with systolic and 12 patient with diastolic CHF were enrolled in the study. Leave-one-out cross validation method was followed and the best accuracy was achieved using multi-layer neural network.

Shah et al. 2015 [28] focused on the distinction of HFpEF subtypes. They employed 397 HFpEF patients and performed detailed clinical, laboratory, electrocardiographic phenotyping of the participating patients. The extracted 67 continuous variables were given as input to statistical learning algorithms (*e.g.* unbiased hierarchical cluster analysis) and penalized model-based clustering. The analysis revealed 3 distinct pheno-groups in terms of clinical characteristics, cardiac structure and function, hemodynamics and outcomes.

A short presentation of the methods for HF subtype classification is presented in Table 3.

## 4 Severity Estimation of HF

Due to the fact that HF is asymptomatic in its first stages, early assessment of the severity of HF becomes a crucial task. The most commonly employed classifications for HF severity are NYHA and ACC/AHA stages of HF. NYHA is based on symptoms and physical activity, while ACC/AHA describes HF stages based on structural changes and symptoms [6]. The two assessment methods provide useful and complementary information about the presence and severity of HF. More specifically, ACC/AHA stages of HF emphasize the development and progression of HF, whereas NYHA focus on exercise capacity of the patient and the symptomatic status of the disease [1].

NYHA classification has been criticized due to the fact that it is based on subjective evaluation and thus intra-observer variability can be introduced [29]. According to the HF guidelines, an objective evaluation of the severity of HF can be provided by the combination of a 2-D ECG with Doppler flow [1]. For the estimation of the severity of HF in the acute setting after myocardial infarction, KILLIP classification can be utilized [1].

Studies reported in the literature, address HF severity estimation through the utilization of machine learning techniques. Specifically, HF severity estimation is expressed either as a 2 or 3 class classification problem, depending on the merge of the NYHA class that has been performed. Akinyokun et al. 2009 [30] proposed a neuro-fuzzy expert system for the severity estimation of HF. A multilayered feed -forward neural network was trained taking as input data from patients from three hospitals in Nigeria. For each patient, seventeen variables were recorded. A measure of significance of each input variable to the output is computed in order redundant information to be removed. Through this procedure six variables, expressing signs and symptoms of HF, were retained and the neural network was retrained using the selected variables. Fuzzy rules were then extracted from the trained datasets. The fuzzy-logic system employs the root mean square error method for drawing inference. The output of the neuro-fuzzy engine is given as input to the decision support engine aiming to optimize the final decision value. The decision support engine carries out the cognitive and emotional filter that corresponds to the objective and subjective feelings, respectively, of the practitioner supporting him/her to make judgments and take decisions regarding the final diagnosis. The cognitive filter average value is added to the neuro-fuzzy values and the decision support intermediate value (DSIV) is computed. The DSIV is then added to the emotional filter average value and the decision support final value (DSFV) is extracted. If DSFV is lower than 0.2, then no HF is presented. If DSFV is > 0.2 and lower or equal to 0.4, then the patient is characterized as mild HF. If DFSV is > 0.4 and lower or equal to 0.7, then the degree of severity is considered to be moderate. In order the patient to be classified to the severe HF class, the DFSV must be between 0.7 and 1. Finally, in case DFSV is > 1, the patient's status is in a very severe condition.

Guidi et al. 2012 [31] developed a computer aided telecare system aiming to assist in the clinical decision of non-specialist personnel involved in the management of HF patients. Among the functionalities of the telecare system is the characterization of patients as mild, moderate or severe. In order this to be achieved, NN, SVM, decision tree and fuzzy expert system classifiers were employed. The classifiers were trained and tested using anamnestic (age, gender, NYHA class) and instrumental data (weight, systolic blood pressure, diastolic blood pressure, EF, BNP, heart rate, ECG parameters (atrial fibrillation, left bundle branch block, ventricular tachycardia))corresponding to 100 (training set) and 36 (testing set) patients, respectively. The distribution of patients to the three severity classes is 35 mild, 31 moderate and 34 severe in the training phase and 15 mild, 8 moderate and 13 severe in the test phase. A 10-fold cross-validation procedure was applied. According to the presented results NN can classify patients with 86.1% accuracy.

Two years later, the same research team [32] enhanced the “pool” of classifiers that were evaluated, with classification and regression tree (CART) and Random Forests. Data from 136 patients, treated by the Cardiology Department of the St. Maria Nuova Hospital (Florence, Italy) were distributed to the three prediction types as follow, 51 mild, 37 moderate and 48 severe. For the evaluation of the classifiers the authors followed a subject based cross validation approach to address the fact that the dataset included cluster-correlated data (baseline and follow-up data of the same patient). More specifically, follow-up data of the same patient were grouped within the same fold. In this way, their assumption that follow-up data spread in a large time period can be considered as separate instances of the dataset, does not affect the independence of the folds. Random Forests outperformed the other methods for the automatic severity assessment. However, the standard deviation was very high. This is due to the fact that in some folds the accuracy was > 90%, while in some others the accuracy was lower than 50%. These folds probably include patients with moderate HF, revealing thus the difficulty of the proposed system in classifying those patients. Although the classification results produced by the CART classifier is 1% lower than those produced by Random Forests, CART algorithm gains the preference of researchers since it can be easily transformed to a set of rules that can be analyzed by medical experts.

Recently the authors of [33] proposed a multi-layer monitoring system for clinical management of CHF. The three layers include the following monitor activities: a) scheduled visits to a hospital following up the presence of a HF event, b) home monitoring visits by nurses, and c) patient's self-monitoring at home through the utilization of specialized equipment. For the activities of the first two layers, a decision support system was developed providing prediction of decompensations and assessment of the HF severity. Random Forests algorithm was employed based on its performance in the studies reported previously. It was evaluated in terms of accuracy, sensitivity and specificity for each class *versus* all the other classes in a 10-fold cross validation. The obtained accuracy was 81.3%, while the sensitivity and specificity were87 and 95%, respectively for class 3 (severe HF *vs.* other). Class 1 (mild HF *vs.* other) was identified with 75% sensitivity and 84% specificity and class 2 (moderate HF *vs.* other) was identified with 67% sensitivity and 80% specificity.

Taking into consideration the fact that ECG provides an objective evaluation of the severity of HF, researchers studied the relationship of long and short-term HRV measures with NYHA class [34], [35], [36], [37], [38] and their discrimination power for HF detection [11], [12]. Pecchia et al. 2011 [39] presented a remote health monitoring system for HF, which provides estimation of HF severity through the utilization of a CART method. HRV measures, extracted from ECG signals, were utilized in order the subject detected with HF to be classified as mild (NYHA I or II) or severe (NYHA III). Different trees were trained using different combinations of the short-term HRV measures. The achieved accuracy, sensitivity, specificity and precision was79.31, 82.35, 75.00 and 82.35%, respectively. The dataset included 83 subjects, 54 control and 29 patients. The 29 patients were distributed to the two classes as follow: 12 were mild and 17 severe.

Two years later, Mellilo et al. 2013 [40] based on the long-term HRV measures and the CART algorithm in order to individuate severity of HF. The classifier separated low risk patients (NYHA I or II) from high risk patients (NYHA III or IV). The HRV measures were extracted from two Holter monitor databases (Congestive Heart Failure RR Interval Database and BIDMC Congestive Heart Failure Database) [17] and corresponded to 12 low risk and 34 high risk patients. However, only 11 low risk and 30 high risk patients were enrolled in the study. The CART algorithm was modified in order to incorporate a feature selection algorithm addressing the issues of small and unbalanced dataset. The results of their method were compared with the results of other classifiers, such as simple CART, C4.5, and Random Forests. All the algorithms were evaluated with and without the application of SMOTE algorithm. The accuracy, precision, sensitivity and specificity of the proposed CART algorithm was 85.40, 87.50, 93.30 and 63.60%, respectively. As mentioned previously, the tree that is created by the CART algorithm can be easily transformed to rules, in the specific case rules for severity estimation. According to the authors the extracted rules were consistent with previous findings. Shahbazi et al. 2015 [41] exploited long- erm HRV measures to estimate the severity of HF and more specifically to classify patients to low risk and high risk. Generalized Discriminant Analysis was applied for reducing the number of features, as well as to overcome overlapping of the samples of two classes in the feature space. The selected features were given as input to a *k*-NN classifier providing classification accuracy 97.43% in the case when both linear and nonlinear features were utilized and 100% accuracy in the case when only nonlinear features were utilized.

Yang et al. 2010 [19] proposed a scoring model allowing classification of a subject to three groups; health group (without cardiac dysfunction), HF-prone group (asymptomatic stages of cardiac dysfunction) and HF group (symptomatic stages of cardiac dysfunction). SVM was employed and the total accuracy was 74.40%. The accuracy for each one of the three groups was 78.79% for healthy group, 87.50% for HF-prone group and 65.85% for the HF group. In total, 289 subjects participated in the study among which 70 were healthy, 59 belonged to HF-prone group (NYHA I, ACC/AHA B-C) and 160 belonged to HF group (NYHA II-III, ACC/AHA C-D). In order imputation of missing values to be achieved, the Bayesian principal components analysis was employed [42]. The decision value of SVM (*v*) [43] is mapped to a specific range in order a definite score to be produced. For this purpose a tan-sigmoid function is applied given by:

(1) $$y=4/\left(1+\exp\left(-4*v\right)-2\right),$$

where *y* is the mapped value. The determination of the cutoff points is achieved using Youden's index [44].

Sideris et al. 2015 [45] proposed a data driven methodology for the estimation of the severity of HF that relies on a clustering-based, feature extraction approach. The authors exploited disease diagnostic information and extracted features. In order to reduce the dimensions of diagnostic codes they identified the disease groups with high frequency of co-occurrence. The extracted clusters were utilized as features for the estimation of severity of the condition of HF patients by employing an SVM classifier. The results were compared with those produced giving as input to the SVM classifier the cluster-based feature set enhanced with the Charlson comorbidity score and an accuracy improvement of up to 14% in the predictability of the severity of condition was achieved. The procedure was applied for each one of the extracted six daily threshold-based outcome variables (I1–I6) labeling the severity of the condition, especially in the context of remote health monitoring.

A short review of the methods addressing HF severity estimation are presented in Table 4.

It must be mentioned that according to the authors knowledge, the HF severity estimation has not been addressed in the past as a four class classification problem (NYHA I, NYHA II, NYHA III, NYHA IV).

## 5 Prediction of Adverse Events

As already mentioned in the Introduction section, HF is a major health problem associated with the presence of serious adverse events, such as mortality, morbidity, destabilizations, re-hospitalizations, affecting both the individuals (*e.g.* reduced quality of life) and the society (*e.g.* increased healthcare costs). The early prediction of those events will allow experts to achieve effective risk stratification of patients and to assist in clinical decision making. Prognostic information could guide the appropriate application of monitoring and treatment, resulting in improvements in the quality of care that is provided, as well as in the outcome of patients hospitalized with HF.

Toward this direction the prediction ability of different factors related to HF morbidity, mortality, destabilizations and re-hospitalizations had been studied. Furthermore, models taking into account simultaneously multiple factors have been reported in the literature using statistical methods (*e.g.* multi-variable Cox regression models). This multi-variable statistical analysis lead to the formation of scores used in clinical practice, providing estimation of risk for mortality (*e.g.* Heart Failure Survival Score [46], Get With the guidelines score [47], Seattle Heart Failure Model [48], EFFECT [49]), re-hospitalizations [50] and morbidity [51].

### 5.1 Destabilizations

Although HF is a chronic syndrome, its evolution does not happen gradually. Alternating periods of relative stability and acute destabilizations exist. The goal of the experts is to predict and prevent destabilizations and death of the HF patient during a stable phase.

Candelieri et al. 2008 [52] adopted Knowledge Discovery (KD) approaches to predict if a patient with CHF in stable phase will further decompensate. A group of 49 CHF patients recurrently visited by cardiologists, every two weeks, was used for the evaluation of the KD approaches. A set of different clinical parameters, selected from guidelines and clinical evidence-based knowledge were evaluated by the cardiologist during the visit, general information and monitored parameters were measured for each patient. Decision trees, Decision Lists, SVM and Radial Basis Function Networks were employed and the leave-patient-out approach was followed to evaluate the performance of the generated models. Decision trees outperformed the other approaches. It provided prediction accuracy 92.03%, sensitivity 63.64%, and False Positive Rate 6.90%. In 2009 Candelieri et al. [53] examined how decision trees and SVM, developed in their previous work, perform on an independent testing set. The results indicated that SVM are more reliable in predicting new decompensation events. The value of evaluation measures is 97.37% accuracy, 100.00% sensitivity, and 2.78% False Positive Rate. Based on this observation they further extended their research activity, by proposing the SVM hyper-solution framework [54]. The term “hyper-solution” is used to describe SVM based on meta-heuristics (Tabu-Search and Genetic Algorithm) searching for the most reliable hyper-classifier (SVM with a basic kernel, SVM with a combination of kernel, and ensemble of SVMs), and for its optimal configuration. The Genetic Algorithm-based framework has been proven more accurate on minority class than the Tabu-Search.

The prediction of the destabilization of HF patients was also addressed by Guidi et al. 2014 [32] and Guidi et al. 2015 [33]. They made a prediction of the frequency (none, rare or frequent) of CHF decompensation during the year after the first visit using five machine learning techniques (NN, SVM, Fuzzy -Genetic Expert System, Random Forests and CART). In Guidi et al. 2014 [32], CART algorithm produced the best classification results (87.6% accuracy). However, in terms of critical error the best results were produced by the Random Forest algorithm. In Guidi et al. 2015 [33], the prediction was addressed as three different classification problems, none *vs.* all, rare *vs.* all and frequent *vs.* all, employing the Random Forests algorithm. The overall accuracy produced by the 10-fold cross-validation procedure is 71.90%, while the sensitivity and specificity for each case that was studied is 57% and 79% for the first case, 65% and 60% for the second case and 59% and 96% for the third case.

A short review of the methods addressing prediction of destabilizations are provided in Table 5.

### 5.2 Re-Hospitalizations

Re-hospitalizations gain the interest of researchers due to their negative impacts on healthcare systems' budgets and patient loads. Thus, the development of predictive modeling solutions for risk prediction is extremely challenging. Prediction of re-hospitalizations was addressed by Zolfaghar et al. 2013 [55], Vedomske et al. 2013 [56], Shah et al. 2015 [28], Roy et al. 2015 [57], Koulaouzidis et al. 2016 [58], Tugerman et al. 2016 [59], and Kang et al. 2016 [60].

Zolfaghar et al. 2013 [55] studied big data driven solutions to predict risk of readmission for CHF within a period of 30-days. Predictive factors were first extracted from the National Inpatient Dataset (NIS) and augmented with the Multicare Health System (MHS) patient dataset. Data mining models, such as logistic regression and Random Forests, were then applied. The best prediction accuracy is 78.00%. The dataset where the prediction models were evaluated contained 15,696 records. In order the authors to examine how the application of big data framework outperforms the traditional systems, when the size of the training set increases, they scaled up the original data linearly several times. Five scenarios of data size were created and the Random Forests algorithm was employed. Among the scenarios, the best prediction accuracy was 87.12%.

Vedomske et al. 2013 [56] applied Random Forests to administrative claims data in order to predict readmissions for CHF patients within 30 day. The data were retrieved from the University of Virginia Clinical Database Repository (CDR) maintained by the Department of Public Health Sciences Clinical Informatics Division. Different variations of the Random Forests classifier were developed depending on the input. More specifically, datasets including procedure data, diagnosis data, a combination of both, and basic demographic data were extracted. The procedure was applied two times; one without prior weighting on the response variable and then with prior weighting aiming to address the issue of imbalanced classes. The discriminative power of the models was measured with the AUC after randomly splitting the datasets into 2/3 training set and 1/3 testing set.

Shah et al. 2015 [28], as previously described (Section 3), detected three HFpEF pheno-groups. Furthermore they studied the association of those groups with adverse outcomes (HF hospitalization, cardiovascular hospitalization, death and combined outcome of cardiovascular hospitalization or death). The results indicated that the created pheno-groups with differential risk profiles provided better discrimination compared to clinical parameters (*e.g.*, the MAGGIC risk score) and B-type Natriuretic Peptide. Additionally, they utilized SVM to predict clinical outcome. Each outcome was coded as binary and 46 phenotypic predictors were included. Radial and sigmoid basis functions were evaluated. The tuning of the values of the gamma and cost parameters was achieved using a derivation cohort of 420 patients, and the evaluation of the performance was performed using a validation cohort including 107 patients. Area under the receiver operating characteristic curve (AUROC), sensitivity, mean specificity, and mean precision were the evaluation measures employed.

Roy et al. 2015 [57] addressed the problem of estimation of readmission risk as a binary classification task. The objective was to identify patients with CHF who are likely to be readmitted within 30 days of discharge (30 days = 1 patient will be readmitted, 30 days = 0 patient will not be readmitted). A dynamic hierarchical classification was followed. The prediction problem was divided in several stages or layers, creating thus a hierarchy of classification models. At each stage-layer the risk of readmission was predicted within certain days (cutoffs). Thus at each stage-layer a binary classification problem was addressed. The output from each layer was combined in order the overall 30-day risk of readmission to be predicted. The method was evaluated on the Washington State Inpatient Dataset^3^ and the Heart Failure cohort data from Multi Care Health Systems (MHS).^4^Logistic regression, Random Forests, Adaboost, Naïve Bayes and SVM classifiers were tested at each layer of dynamic hierarchical classification framework. The best classifier at each stage was determined through a 10-fold cross-validation procedure on training set.

Koulaouzidis et al. 2016 [58] used daily collected physiological data such as blood pressure, heart rate, weight, while the patients were at their home and predicted HF patients' re-hospitalization through a Naive Bayes classifier. They assessed, by employing an analysis of vectors, the predictive value of each of the monitored signals and their combinations. They observed that the best predictive results were obtained with the combined use of weight and diastolic blood pressure received during a time period of 8 days (8-day telemonitoring data). The achieved AUROC was 0.82 ± 0.02) allowing the authors to conclude that the telemonitoring has high potential in the detection of HF decompensation, however, the validity of the proposed approach in the clinical management of patients should be examined through a large-scale prospective study.

Kang et al. 2016 [60] like Koulaouzidis et al. 2016 [58] worked with data from telemonitored patients aiming to predict first re-hospitalization during the 60-day home healthcare episode. They utilized the OASIS-C dataset and they employed bivariate analysis for selecting the variables that can act as predictors and lead to the development of the best decision tree model. The J48, using 10-fold cross-validation procedure, was used to create the decision tree. 67% of the dataset was used for the construction of the tree, while 33% was used for its validation. True Positive Rate, the False Positive Rate and the AUROC are employed as evaluation measures.

Tugerman et al. 2016 [59], in order to predict hospital readmissions within 30 days following discharge, combined the C5.0 and SVM classifiers controlling thus the trade-off between reasoning transparency and predictive accuracy. Once they optimized the two classifiers, the optimization of the mixed model was followed. In order the two models (C5.0 and SVM) to be combined a tree confidence threshold was predefined. Records that are predicted with tree confidence below the predefined one are further classified by SVM. The performance of the mixed model was measured in terms of sensitivity, specificity, F1 score, positive predictive values (PPV), negative predictive values (NPV). Different threshold values were employed for the testing and training set.

Table 6 presents a short review of the literature regarding prediction of re-hospitalizations.

### 5.3 Mortality

HF is one of the leading causes of death worldwide. Accurate HF survival prediction models can provide benefits both to patients and physicians, with the most important being the prevention of such an adverse event.

Besides Shah et al. 2015 [28], Fonarrow et al. 2005 [61] estimated mortality risk in patients hospitalized with acute decompensated heart failure (ADHF), Bohacik et al. 2013 [62] applied an alternating decision tree to predict risk of mortality within six months for heart failure patients and two years later [63] they present a model based on fuzzy logic, Panahiazar et al. 2015 [64] exploited data from electronic health records of the Mayo Clinic and they performed HF survival analysis using machine learning techniques. One year later, the same research team [65] applied Contrast Pattern Aided Logistic Regression (CPXR(Log)) with the probabilistic loss function to the same dataset, developing and validating prognostic risk models to predict 1, 2, and 5 year survival in HF. Austin et al. 2012 [66] and Subramanian et al. 2011 [67] predicted 30 day and 1 year mortality, respectively by employing ensemble classifiers. Finally, Ramirez et al. 2015 [68] addressed the problem of mortality prediction as a classification problem where the classes are Sudden cardiac death (SCD), Pump failure Death (PFD) and Non cardiac death, survivors. The following classification problems were studied: i) SCD *vs.* the rest, ii) PFD *vs.* the rest and iii) SCD victims, PFD victims and others (non-CD and survivors).

Fonarrow et al. 2005 [61] developed a risk stratification model for predicting in-hospital mortality exploiting the Acute Decompensate Heart Failure National Registry (ADHERE) of patients hospitalized with a primary diagnosis of ADHF in 263 hospitals in the United States [69] and utilizing the CART classification algorithm. The data included in the ADHERE registry were divided in two cohorts. More specifically, the first 33,046 hospitalizations (derivation cohort) were analyzed to develop the model, while data from 32,229 subsequent hospitalizations (validation cohort) were employed in order the validity of the model to be tested. From 39 variables, selected out of 80 included in the ADHERE registry, blood urea nitrogen, systolic blood pressure, levels of serum are identified as predictors for in hospital mortality. The CART tree was able to stratify patients into high, intermediate and low risk.

Bohacik et al. 2013 [62] classified 2023 patients diagnosed with HF into two possible predictions, alive or dead. Nine features describe the instance of patients expressing information regarding pulse rate, NT-proBNP level, blood sodium level, blood uric acid level, blood creatinine level, weight, height, gender and age. In order classification to be achieved an alternating decision tree, which maps each HF patient to a real valued prediction, was utilized. The prediction is the sum of the predictions of the base rules in its set, while the classification is the sign of the prediction. The achieved sensitivity is 37.31%, specificity is 91.53%, positive predictive value is 60.25%, negative predictive value is 80.94% and accuracy is 77.66%.

Two years later, Bochacik et al. 2015 [63] presented a model for the estimation of risk mortality within 6 months employing ambiguity and notions of fuzzy logic. The model stores knowledge for the patients in the form of fuzzy rules and classifies a patient to dead or alive using those rules. The authors compared the results of the proposed classifier with those produced by the application of a Bayesian network classifier, a nearest neighbor classifier, multilayer neural network, 1R classifier, a decision list, and a logistic regression model. Furthermore, the authors evaluated the interpretability using measures expressing the complexity of the fuzzy rules (average rule length, average number of rules, and average, minimal and maximal number of assignments in the conditions of rules).

Panahiazar et al. 2015 [64] applied decision trees, Random Forests, Adaboost, SVM and logistic regression to a dataset produced by the electronic health records of the Mayo Clinic. The dataset initially included 119,749 patients admitted to the Mayo Clinic from 1993 to 2013. 842 patient records were excluded due to incomplete and missing data and some others because they did not met the criteria defined by the experts. Thus, a final cohort with 5044 HF patients was used. For each patient 43 predictor variables, expressing demographic data, vital measurements, lab results, medication and co-morbidities, were recorded. The class variable corresponded to mortality status, consequently three versions of the dataset were created, each one corresponding to survival period (1-year, 2-year, 5-year). 1560 instances out of 5044 were used for training and the rest 3484 instances for testing. The predictor variables were divided into two sets, one including the same variables with those used in Seattle Heart Failure Model (baseline set) and one including the predictors of the first set plus race, ethnicity, body mass index, calcium channel blocker and 26 different co-morbidities (extended set). The above mentioned classifiers were applied to baseline and extended the set for 1-year, 2-years and 5-years prediction models. The authors observed that logistic regression and Random Forests were more accurate models compared to others, as well as that the incorporation of the 26 co-morbidities improves the results.

Taslimitehrani et al. 2016 [65] employed the CPXR(Log) classification algorithm with the probabilistic loss function to the cohort of 5044 patients described previously. The authors compared the results of CPXR(Log) classification algorithm with the results produced by decision trees, Random Forests, Adaboost, SVM and logistic regression. The CPXR(Log) classification algorithm outperformed the other classifiers and the prediction accuracy was 93.70% for 1 year mortality, 83.00% for the 2 years mortality and 78.60% for the 5 years mortality. The CPXR algorithm uses a pattern as logical characterization of a subgroup of data, and a local regression model characterizing the relationship between predictor and response for data of that subgroup. In case the patient's data match to one of the patterns, then the local model was built for the specific group of patients instead of the baseline model that was built for the whole population is used. According to the authors, the analysis of those patterns revealed the heterogeneity of HF between the patients. In order this heterogeneity to be taken into consideration for the survival prediction, the utilizations of the local models and different patterns is recommended.

Subramanian et al. 2011 [67] focused on predicting the mortality within 1 year by building logistic regression models and ensemble models that incorporate time-series measurements of biomarkers such as cytokine. More specifically, three logistic regression models were built to predict survival beyond 52 weeks after entry into the trial. The models are differentiated depending on the input they receive. The first model uses standard baseline measurements, allowing the experts to compare their results with those reported in the literature, the second model incorporates baseline measurements and baseline cytokine evaluating thus the contribution of cytokines to the prediction of survival and the third model includes cytokine measurements up to week 24 to the second set of predictor variables assessing thus the utility of serial follow-up measurement to predict survival. The ensemble model was built by combining the three models previously described. The final classification of the subjects as a survivor or non-survivor is determined through a majority voting procedure.

Austin et al. 2012 [66] reduced the prediction horizon of mortality to one month. In order the prediction to be achieved ensemble-based methods, including bootstrap aggregation (bagging) of regression trees, random forests, and boosted regression trees were employed. The method was evaluated in two large cohorts of patients hospitalized with either acute myocardial infarction (16.230 subjects) or congestive heart failure (15.848 subjects) and the best results were produced by logistic regression trees.

Ramirez et al. [68] employed the SVM classifier and holter ECG recordings for 597 CHF patients with sinus rhythm enrolled in the MUSIC study to classify them to sudden cardiac death victims, pump failure death victims and other (the latter including survivors and victims of non-cardiac causes). According to the specific study, the ECG risk marker quantifying the slope of the T-peak-to-end/RR regression, T-wave alternans and heart rate turbulence slope can act as discriminators of the classes mentioned above.

Table 7 presents a short review of the literature regarding prediction of mortality.

## 6 Summary and Outlook

HF is a chronic disease characterized by a variety of unpleasant outcomes, such as poor QoL, recurrent hospitalization, high mortality and significant cost burden. A significant deterrent of the above mentioned serious consequences is early diagnosis of HF (detection of HF, estimation of the etiology and severity of HF), as well as early prediction of adverse events. Toward this direction the application of machine learning techniques contributed significantly. Researchers applied data mining techniques in order to address issues concerning management of HF either separately or in combination. More specifically, detection of HF is based mainly on the utilization of HRV measures in combination with classifiers such as SVM, CART and *k-*NN. The studies either utilize short-term HRV measures or long-term HRV measures. None of the studies has attempted to compare or combine short- and long-term HRV measures. However, there are studies that incorporate, in the classification process, data expressing the results of clinical examination, presenting symptoms, lab tests *etc*.The utilization of different sources of data in each one of these studies limits their comparison, unlike the methods that detect HF, by utilizing HRV measures, that is applied to publicly available datasets commonly used in all studies. After the detection of HF, the estimation of the etiology or the characterization of the type of HF follows. Different classifiers were applied in order to classify a patient into one of the two major HF subtypes (HFpEF *vs.* HFrEF). All the studies addressed the issue as a two class classification problem and did not take into consideration the patients belonging to the so called “gray zone” (HFmrEF). Only Betanzos et al. 2015 [19] included in their study this group of patients. However, they did not consider patients with HFmrEF as a separate group (3 class classification problem) but included them in one of the two major HF types by setting different cutoff points. The next step in the management of HF concerns the estimation of its severity. According to the studies reported in the literature, the problem of HF severity estimation is transformed to a two or three class classification problem. The patient's status is characterized as mild, moderate or severe. The definition of those characterizations is differentiated between the studies. For example, in some studies the characterization “severe” refers to patients belonging to NYHA class III or IV, while in some other only patients belonging to NYHA class IV are included. Furthermore, according to the authors' knowledge, no one have tried to classify the patients into 4 NYHA classes. Finally, prediction of adverse events has been attempted by the researchers. Models predicting destabilizations, re-hospitalizations, and mortality have been presented in the literature. The time frame of prediction depends on the adverse events. However, the interest of the researchers has turned to the prediction of HF since the earlier HF is detected, the more likely change health outcomes for people can be achieved. Wu et al. 2010 [18] and Aljaaf et al. 2015 [2] presented their work regarding the specific issue, with the work of Aljaaf et al. 2015 [2] achieving the best prediction accuracy. Recently a research team from Sutter Health, a Northern California not-for-profit health system, and the Georgia Institute of Technology, have proposed a method that according to the authors has the potential to reduce HF rates and possibly save lives since it can predict disease onset nine months before doctors can now deliver the diagnosis.^5^ The method employs deep learning, a branch of machine learning based on learning representations of data. Deep learning has been applied to problems such as computer vision and speech understanding. In the future the application of deep learning to personalized prescriptions, therapy recommendation, clinical trial recruitment, tasks involving prediction and detection of disease will be studied, opening a new window in the management of the HF and other diseases. The current work provides a comprehensive review and comparison (Table 8), in terms of advantages and disadvantages, of the methods reported in the literature that address, either separate or in combination, all the aspects of the HF management employing machine learning techniques.

## Figures and Tables

**Table 1:** HF detection methods using HRV measures - review of the literature.

Authors	Method	Data	Features	Evaluation measures
Asyali et al. 2003 [7]	Linear discriminant analysis Bayesian classifier	No. of data 54 normal subjects 29 patients with CHF	Predictor features Long-term HRV measures	Observed Agreement Rate:93.24%, Sensitivity (true positive):81.82% Specificity (true negative): 98.08% kappa statistics: 0.832 (95% confidence interval: 0.689–0.974)
		Source of data RR interval databases at PhysioBank include beat annotation files for long-term (∼ 24 h) ECG recordings	Response feature Normal CHF	Validation
				n/a
Isler et al. 2007 [8]	Feature selection (genetic algorithm) Minmax Normalization *k*-NN	No. of data 54 normal subjects 29 CHF subjects	Predictor features Short-term HRV measures + Wavelet entropy measures	*k* = 5 Sensitivity:96.43% Specificity:96.36% Accuracy: 96.39% *k* = 7 Sensitivity:100% Specificity:94.74% Accuracy: 96.39%
		Source of data RR interval records at the MIT/BIH database include beat annotation files for long-term (∼ 24 h) ECG recordings	Response feature Normal CHF	Validation
				Leave-one-out cross-validation
Thuraisingham 2009 [9]	Features from difference plot second order (SODP) *k*-NN	No. of data 36 normal subjects 36 CHF subjects	Predictor features Central tendency measure standard deviation of the RR intervals D (distance)	Success rate: 100%
		Source of data The RR interval data was obtained from MIT-BIH Normal Sinus Rhythm database, BIDMC congestive Heart Failure database, and congestive heart failure RR interval database	Response feature Normal CHF	Validation
				n/a
Elfadil et al. 2011 [10]	Supervised Multi-layer perceptron	No. of data Training 53 Normal sinus rhythm (NSR) & 17 CHF recordings Testing 12 CHF and 12 normal subjects	Predictor features Power spectral density R1 (band 1), R2 (bands: 2 to 3), R3 (bands: 4 to 10), R4 (bands: 11 to 16), R5 (bands: 17 to 24), R6 (bands: 25 to 32).	Sensitivity: 85.30% Specificity: 82.00% Accuracy: 83.65%
		Source of data Data randomly simulated from Massachusetts Institute of Technology (MIT) database	Response feature Normal CHF	Validation
				Testing 12 CHF and 12 normal subjects
	Unsupervised Normalization Self-organizing map	No. of data Training 1000 CHF &1000 normal simulated randomly from 17CHF and 53 normal subjects Testing 1000 CHF &1000 normal simulated randomly from 12 CHF and 12 normal subjects	Predictor features Power spectral density R1 (band 1), R2 (bands: 2 to 3), R3 (bands: 4 to 10), R4 (bands: 11 to 16), R5 (bands: 17 to 24), R6 (bands: 25 to 32).	Sensitivity: 89.10% Specificity: 96.70% Accuracy: 92.90%
		Source of data Massachusetts Institute of Technology (MIT) database	Response feature Normal CHF	Validation Testing 1000 CHF &1000 normal simulated randomly from 12 CHF and 12 normal subjects
Pecchia et al. 2011 [11]	CART with feature selection	No. of data 54 normal subjects 29 CHF subjects	Predictor features Short-term HRV measures	Sensitivity: 89.70% Specificity: 100.00%
		Source of data Normal subjects was retrieved from the Normal Sinus Rhythm RR Interval Database CHF group was retrieved from the Congestive Heart Failure RR Interval Database	Response feature Normal CHF	Validation
				Leave-one-out cross-validation
Mellilo et al. 2011 [12]	CART with feature selection	No. of data 72 normal subjects 44 CHF subjects	Predictor features Long-term HRV measures	Sensitivity: 89.74% Specificity: 100.00%
		Source of data Normal subjects were retrieved from the Normal Sinus Rhythm RR Interval Database and from the MIT-BIH Normal Sinus Rhythm Database The data for the CHF group were retrieved from the Congestive Heart Failure RR Interval Database and from the BIDMC Congestive Heart Failure Database	Response feature Normal CHF	Validation
				10 fold-cross-validation
Jovic et al. 2011 [13]	SVM, MLP, C4.5, Bayesian classifiers	No. of data 25 normal subjects 25 CHF subjects	Predictor features Correlation dimension, Spatial filling index, Central tendency measure, Approximate entropy (four features), Standard deviation of the NN (or R-R) interval – SDNN, root of the mean squared differences of N successive R-R intervals – RMSSD, ratio of the number of interval differences of successive R-R intervals that are greater than 20 ms and the total, number of R-R intervals - pNN20, HRV triangular index	SVM Sensitivity: 77.2% Specificity: 87.4% MLP Sensitivity: 96.6% Specificity: 97.8% C4.5 Sensitivity: 99.2% Specificity: 98.4% Bayesian Sensitivity: 98.4% Specificity: 99.2%
				Validation
		Source of data BIDMC congestive heart failure database MIT-BIH normal sinus rhythm database Normal sinus rhythm RR interval database	Response feature Normal CHF	10 × 10-fold-cross-validation
Yu et al. 2012 [14]	Feature selection (UCIMFS, MIFS, CMIFS, mRMR) SVM	No. of data 54 normal subjects 29 CHF subjects	Predictor features Long-term HRV measures + Age and Gender	All features Sensitivity: 93.10% Specificity: 98.14% Accuracy: 96.38% UCMIFS Sensitivity: 96.55% Specificity: 98.14% Accuracy: 97.59% MIFS Sensitivity: 93.10% Specificity: 98.14% Accuracy: 96.38% CMIFS Sensitivity: 93.10% Specificity: 100.00% Accuracy: 97.59% mRMR Sensitivity: 93.10% Specificity: 98.14% Accuracy: 96.38%
		Source of data Congestive heart failure (CHF) and normal sinus rhythm (NSR) database, both of which were available on the PhysioNet	Response feature Normal CHF	Validation
				Leave-one-out cross-validation
Yu et al. 2012 [15]	Feature selection by Genetic Algorithm (GA) SVM	No. of data 54 Normal subjects 29 CHF subjects	Predictor features Bispectral analysis based features	RBF kernel Sensitivity: 95.55% Specificity: 100% Linear kernel Sensitivity: 93.10% Specificity: 98.14%
		Source of data Data for the research were provided by the congestive heart failure (CHF) database (chf2db) and normal sinus rhythm (NSR) database (nsr2db), both of which are available on the PhysioNet	Response feature Normal CHF	Validation
				Leave-one-out cross validation
Liu et al. 2014 [16]	Feature selection Feature normalization Feature combination SVM & *k*-NN	No. of data 30 normal subjects 17 CHF subjects	Predictor features Short-term HRV measures	SVM Accuracy: 100.00% Precision: 100.00% Sensitivity: 100.00% *k*-NN Accuracy: 91.49% Precision: 94.12% Sensitivity: 84.21%
		Source of data Normal subjects was retrieved from the Normal Sinus Rhythm RR Interval Database CHF group was retrieved from the Congestive Heart Failure RR Interval Database	Response feature Normal CHF	Validation
				Cross-validation
Narin et al. 2014 [17]	Filter based backward elimination feature selection SVM, *k*-NN, LDA, MLP, RBF classifier	No. of data 54 normal subjects 29 CHF subjects	Predictor features Short term HRV measures + Wavelet transform measures	SVM Sensitivity: 82.75% Specificity: 96.29% Accuracy: 91.56% *k*-NN *k* = 5 Sensitivity: 65.51% Specificity: 96.29% Accuracy: 85.54% Polynomial LDA Sensitivity: 75.86% Specificity: 90.74% Accuracy: 85.54% MLP Sensitivity: 82.75% Specificity: 92.59% Accuracy: 89.15% RBF Sensitivity: 58.62% Specificity: 96.29% Accuracy: 93.13%
		Source of data The data used in this study were obtained from the normal sinus rhythm and congestive heart failure RR interval databases from the MIT/BIH database in PhysioNET	Response feature Normal CHF	Validation
				Leave-One-Out cross-validation
Heinze et al. 2014 [18]	Feature extraction by Power spectral density(PSD) Conventional spectral analysis Ordinal patterns Learning Vector Quantization (LVQ) classifier	No. of data 54 Normal subjects 29 CHF subjects	Predictors features HRV measures	PSD features 13.6% error at 50 min Conventional analysis features 17.5% error at 60 min Ordinal patterns 18% error at 45 min
		Source of data Normal sinus rhythm and congestive heart failure RR interval databases from the MIT/BIH database in PhysioNET	Response feature Normal CHF	Validation
				Multiple-hold-out validation (80% training data, 20% testing data) with 50 repetitions

CHF: Congestive Heart Failure, CART: Classification and Regression Tree, UCMIFS: Uniform Conditional Mutual Information Feature Selection CMIFS: Conditional Mutual Information Feature Selection, MIFS: Mutual Information Feature Selection, mRMR: min-redundancy max-relevance, SVM: Support Vector Machines, *k*-NN: *k* Nearest Neighbors, RBF: Radial Basis Function, MLP: Multi-Layer Perceptron, LDA: Linear Discriminant Analysis, HRV: Heart Rate Variability.

**Table 2:** HF detection methods not using HRV measures - review of the literature.

Authors	Method	Data	Features	Evaluation measures
Yang et al. 2010 [19]	Scoring model using SVM	No. of data 153 subjects 65 HF subjects, 30 HF-prone subjects 58 healthy subjects	Predictor features parameters are selected from clinical tests, *i.e.*, blood test, heart rate variability test, echocardiography test, electrocardiography test, chest radiography test, 6 min walk distance test and physical test	SVM model 1 Sensitivity: 75% Specificity: 94% Youden's index: 69% SVM model 2 Sensitivity: 100% Specificity: 80% Youden's index: 80%
		Source of data Data collected at Zhejiang Hospital	Response feature Non-HF group (Healhty group or HF-prone group) HF group	Validation
				90 subjects used as test cases
Gharehchopogh et al. 2011 [20]	Neural networks	No. of data 40 subjects 26 normal subjects 14 HF subjects	Predictor features Gender, age, blood pressure, smoking habit	Training set True positive rate: 95.00%, False positive rate: 9.00%, Precision: 95.00%, Recall: 95.00%, F-measure: 94.00% AUC: 95%. Testing set Percentage prediction: 85%
		Source of data Data collected at referral health center in one of region in Tabriz	Response feature HF yes HF no	Validation
				Testing set
Son et al. 2012 [4]	Rough sets based decision model Logistic regression based decision model	No. of data 159 subjects 71 CHF subjects 88 normal subjects	Predictor features Laboratory findings	Rough sets based decision model Accuracy: 97.5% Sensitivity: 97.2% Specificity: 97.7% Positive predictive value: 97.2% Negative predictive value: 97.7% Area under ROC curve: 97.5% Logistic regression based decision model Accuracy: 88.7% Sensitivity: 90.1% Specificity: 87.5% Positive predictive value: 85.3% Negative predictive value: 91.7% Area under ROC curve: 88.8%
		Source of data Data collected at the emergency medical center of Keimyung University Dongsan Hospital	Response features Normal CHF	Validation
				10-fold-cross-validation
Masetic et al. 2016 [21]	Random Forests SVM C4.5 ANN *k-*NN	No. of data 15 CHF subjects 13 normal subjects	Predictor features Features extracted by raw ECG using Burg method for autoregressive	BIDMC congestive heart failure + MIT BIH Arrhythmia databases ROC area: 100% F-measure: 100% Accuracy: 100% PTB Diagnostic ECG + MIT BIH Arrhythmia databases ROC area: 100% F-measure: 100% Accuracy: 100%
		Source of data Beth Israel Deaconess Medical Center (BIDMC) Congestive Heart Failure PTB Diagnostic ECG Normal heartbeats were taken from MIT–BIH Arrhythmia database	Response features Normal CHF	Validation
				10-fold cross-validation
Zheng et al. 2015 [24]	Wavelet Transform for Heart Sound signals Least Square Support Vector Machine (LS-SVM) Neural Network Hidden Markov model	No. of data 64 CHF subjects 88 healthy volunteers	Predictor features Heart Sound and Cardiac Reserve features The ratio of diastolic to systolic duration. The ratio of the amplitude of the first heart sound to that of the second heart sound. The width of multifractal spectrum. The frequency corresponding to the maximum peak of the normalized PSD curve. Adaptive sub-band energy fraction shown.	LS-SVM Accuracy: 95.39% Sensitivity: 96.59% Specificity: 93.75%
		Source of data Chongqing University and the First and the Second Affiliated Hospitals of Chongqing University of Medical Sciences	Response feature Normal CHF	Validation
				The double-fold cross-validation

SVM: Support Vector Machines, HF: Heart Failure, CHF: Congestive Heart Failure, ANN: Artificial Neural Networks, ROC: Receiver Operating Characteristic, AUC: Area Under Curve, LS-SVM: Least Square Support Vector Machine, *k-*NN: *k*-Nearest Neighbors.

**Table 3:** Short presentation of the studies reported in the literature addressing HF subtypes classification.

Authors	Method	Data	Features	Evaluation measures				
Austin et al. 2013 [26]	Random Forests	No. of data 3.697 patients for training 4.515 patients for testing	Predictor features Demographic characteristics, vital signs, presenting signs and symptoms, results of laboratory investigations, and previous medical history	Sensitivity: 37.8%	PPV: 69.6%			
				Specificity: 89.7%	NPV: 69.7%			
		Source of data Data collected during the Enhanced Feedback for Effective Cardiac Treatment (EFFECT) study	Response feature HFpEF HFrEF	Validation				
				Testing set of 8.339 subjects				
Betanzos et al. 2015 [25]	SVM PEGASOS	No. of data 48 real patients (35 HFpEF and 13 HFrEF) for training 63 Monte Carlo simulated instances (34 HFpEF and 29 HFrEF) for testing	Predictor features End Systolic Volume Index	Training error %: 2.08	Test error %: 4.76			
		Source of data Clinical study conducted at Cardiovascular Center, OLV Clinic, Aalst, in Belgium	Response feature HFpEF HFrEF	Validation				
				Testing set of 63 instances 10-fold cross-validation				
	SVM PEGASOS	No. of data 48 real patients (35 HFpEF and 13 HFrEF) for training 403 Monte Carlo simulated instances (150 HFpEF, 137 HFrEF, 116 HFmrEF) for testing	Predictor features End Systolic Volume Index	True Positive Rate	40%	45%	50%	55%
				HFpEF	100%	91%	98%	99%
				HFrEF	87%	96%	97%	98%
		Source of data Clinical study conducted at Cardiovascular Center, OLV Clinic, Aalst, in Belgium	Response feature HFpEF HFrEF Including patients belonging to “gray zone”	Validation				
				Testing set of 403 instances				
Isler 2016 [27]	Min-Max Normalization *k*-NN, MLP	No. of data 18 patients with systolic CHF 12 patients with diastolic CHF	Predictor features Short term HRV measures	MPL Sensitivity: 93.75% Specificity: 100% Accuracy: 96.43% *k*-NN Sensitivity: 87.50% Specificity: 91.67% Accuracy: 89.29%				
		Source of data Holter ECG data used in this study were obtained from the Faculty of Medicine in Dokuz Eylul University	Response feature patients with systolic CHF patients with diastolic CHF	Validation				
				Leave-one-out cross-validation				

PPV: Positive Predictive Value, NPV: Negative Predictive Value, MLP: Multi-Layer Perceptron, *k*-NN: *k* Nearest Neighbors, SVM: Support Vector Machines, LS-SVM: Least Square SVM, HF: Heart Failure, CHF: Congestive Heart Failure, HRV: Heart Rate Variability, HFpEF: Heart Failure with preserved Ejection Fraction, HFrEF: Heart Failure with reduced Ejection Fraction, AUC: Area Under Curve.

**Table 4:** Short presentation of the studies reported in the literature addressing HF severity estimation.

Authors	Method	Data	Features	Evaluation measures						
Akinyokun et al. 2009 [30]	Neuro-fuzzy expert system	No. of data 30 subjects	Predictor features Signs and symptoms of heart failure: chest pain, dyspnea (shortness of breath), orthopnea, palpitation, cough, fatigue, tachycardia, cyanosis, edema, nocturia, high blood pressure, low blood pressure, heart rate, rales, (crackles in lungs), elevated neck veins, hepamegaly, wheeze, heart sound, alteration in thought process, changes in level of consciousness, absence of emotion, heart murmur, pleural effusion, pulmonary edema, cardio thoracic ratio, upper zone flow distribution and echocardiogram.	Training set Mean Square Error: 0.021, High Standard Deviation: 0.036, Average minimum normalized mean Square Error: 0.026, Correlation coefficient: 0.988 Overall percentage error: 1.24%, Akaiike Information Criteria (AIC): 171.288 Minimum Description Length: 129.107.						
		Source of data Data collected at three hospital of Nigeria	Response feature Mild HF Moderate HF Severe HF	Validation						
				70% of the datasets were used for training, 20% were employed as testing datasets 10% were used as cross validation datasets.						
Guidi et al. 2012 [31]	Computer aided telecare system NN/SVM/Fuzzy-Genetic/Decision Tree	No. of data 136 subjects 51 mild, 37 moderate, 48 severe	Predictor features Anamnestic data (age, gender, NYHA class) Instrumental data (weight, systolic blood pressure, diastolic blood pressure, EF, BNP, heart rate, ECG parameters (atrial fibrillation, left bundle branch block, ventricular tachycardia))	Accuracy						
				NN		86.10%				
				SVM		69.40%				
				FG		72.20%				
				DT		77.80%				
		Source of data Data collected at the Cardiology Department of the St. Maria Nuova Hospital (Florence, Italy)	Response feature Mild HF Moderate HF Severe HF	Validation						
				100 subjects for training 36 subjects for testing						
Guidi et al.2014 [32]	NN/SVM/Fuzzy-Genetic/CART/Random Forests	No. of data 136 subjects 51 mild, 37 moderate, 48 severe	Predictor features Anamnestic data (age, gender, NYHA class) Instrumental data (weight, systolic blood pressure, diastolic blood pressure, Ejection Fraction (EF), BNP, heart rate, ECG parameters (atrial fibrillation, left bundle branch block, ventricular tachycardia))		Accuracy	Std		Critical errors		
				NN	77.80%	7.4		0		
				SVM	80.30%	9.4		3		
				FG	69.90%	9.9		1		
				CART	81.80%	8.9		2		
				RF	83.30%	7.5		1		
		Source of data Data collected at the Cardiology Department of the St. Maria Nuova Hospital (Florence, Italy)	Response feature Mild HF Moderate HF Severe HF	Validation						
				A person independent ten-fold cross validation						
Guidi et al. 2015 [33]	Multi-layer monitoring system for clinical management of CHF Random Forests	No. of data 250 patients 93 mild, 92 moderate, 65severe	Predictor features Height and weight (Body Mass Index) Systolic and diastolic blood pressure Heart rate Oxygen saturation Ejection fraction (EF) BNP or NT-proBNP Bioelectrical impedance vector (BIVA) parameters NYHA class 12-lead EKG report (e.g., presence of bundle branch block, tachycardia, atrial fibrillation, etc.) Etiology Comorbidity Current therapy, pharmaceutical and surgical (pacemaker or ICD ICD/CRT)	Accuracy: 81.30%						
				“Mild” vs. all Sensitivity: 75.00% Specificity: 84.00%						
				“Moderate” vs. all Sens: 67.00% Spec: 80.00%						
				“Severe” vs. all Sensitivity: 87.00% Specificity: 95.00%						
		Source of data Clinical study data collected through home visits and follow up	Response feature Mild HF Moderate HF Severe HF	Validation						
				10-fold cross-validation						
Pecchia et al. 2011 [39]	Remote health monitoring system for HF CART Mild, Severe	No. of data 54 controls 29 patients 12 mild, 17 severe	Predictor features HRV measures	Accuracy: 79.31% Sensitivity: 82.35% Specificity: 75.00% Precision: 82.35%						
		Source of data Normal subjects was retrieved from the Normal Sinus Rhythm RR Interval Database CHF group was retrieved from the Congestive Heart Failure RR Interval Database	Response feature Mild (NYHA class I or II) Severe (NYHA class III)	Validation						
				Cross-validation						
Mellilo et al. 2013 [40]	1. Proposed CART/ 2. CART/ 3. CART with SMOTE/ 4. C4.5/5. C4.5 with SMOTE/6. RF/7. RF with SMOTE Low risk (NYHA I or II), High risk (NYHA III or IV)	No. of data 11 low risk 30 high risk	Predictor features Long-term HRV measures		Accuracy	Sens	Spec	Precision		
				1	85.40%	93.30%	63.60%	87.50%		
				2	73.20%	100.00%	0.0%	73.20%		
				3	75.00%	73.30%	77.30%	81.50%		
				4	65.90%	73.30%	45.50%	78.60%		
				5	84.60%	93.30%	86.40%	89.30%		
				6	73.20%	86.70%	36.40%	78.80%		
				7	82.70%	83.30%	81.80%	86.20%		
		Source of data Congestive Heart Failure RR Interval Database BIDMC Congestive Heart Failure Database	Response feature Low risk (NYHA class I and II) High risk (NYHA class III and IV)	Validation						
				10-fold cross-validation						
Yang et al.2010 [19]	Scoring model SVM Healthy group, HF-prone group, HF group	No. of data 153 subjects 65 HF subjects, 30 HF-prone subjects 58 healthy subjects	Predictor features parameters are selected from clinical tests, i.e., blood test, heart rate variability test, echocardiography test, electrocardiography test, chest radiography test, six minutes walk distance test and physical test	Total accuracy: 74.40%						
				Accuracy for the healthy group:78.79%						
				Accuracy for the HF-prone group: 87.50%						
				Accuracy for the HF group: 65.85%						
		Source of data Data collected at Zhejiang Hospital	Response feature Healthy group HF-prone group HF group	Validation						
				90 subjects used as test cases						
Shahbazi et al. 2015 [41]	Feature extraction with Generalized Discriminant Analysis (GDA) k-NN	No. of data 12 low risk HF subjects 32 high risk HF subjects	Predictor features Long-term HRV measures	Linear + nonlinear features + GDA Accuracy: 97,43% Precision: 96,66% Sensitivity: 100% Specificity: 90% Nonlinear features + GDA Accuracy: 100% Precision: 100% Sensitivity: 100% Specificity: 100%						
		Source of data Congestive Heart Failure RR intervals Database with patients suffering from CHF (NYHA classes I–III) BIDMC Congestive Heart Failure Database with patients suffering from severe CHF (NYHA class III and IV).	Response feature Low risk HF High risk HF	Validation						
				Leave-one-out cross-validation						
Sideris et al. 2015 [45]	Feature extraction with Hierarchical clustering SVM	No. of data 7 million discharge records 3041 patients	Predictor features Demographics (gender, age, race), diagnostic information encoded in ICD-9-CM and hospitalization specific information including blood test results and discharge diagnoses coded as ICD-9-CM codes.	Alert	Accuracy (%)		TPR (%)		TNR (%)	
				I1	70.72	66.18	64.21	59.74	77.24	72.63
				I2	58.57	51.63	52.65	53.06	64.49	50.20
				I3	73.15	70.73	67.31	64.31	79.00	77.15
				I4	65.48	63.97	71.78	72.74	59.18	55.21
				I5	69.39	69.15	63.66	61.10	75.12	77.20
				I6	67.87	63.16	54.71	52.94	81.03	73.38
		Source of data Training 2012 National Inpatient Sample (NIS), Healthcare Cost and Utilization Project (HCUP) which contains 7 million discharge records and ICD-9-CM codes Testing Ronald Reagan UCLA Medical Center Electronic Health Records (EHR) from 3041 patients	Response feature Low risk High risk	Validation						
				10-fold cross-validation						

NN: Neural Networks, SVM: Support Vector Machines, FG: Fuzzy-Genetic, DT: Decision Tree, RF: Random Forests, Std: Standard deviation, TPR: True Positive Rate, TNR: True Negative Rate, Sens: Sensitivity, Spec: Specificity, HF: Heart Failure, NYHA: New York Heart Association, CART: Classification and regression tree, GDA: Generalized Discriminant Analysis, k-NN: k Nearest Neighbors, SMOTE: Synthetic Minority Over-sampling Technique

**Table 5:** Prediction of destabilizations - short review of the literature.

Authors	Method	Data	Features	Evaluation measures
Candelieri et al. 2008 [52]	Decision trees	No. of data 49 patient with CHF	Predictor features Systolic Blood Pressure (SBP), Heart Rate (HR), Respiratory Rate (RR), Body Weight (weight), Body Temperature (BT) Total Body Water (TBW). Patient condition evaluated by the cardiologist during the visit, Gender, Age, NYHA class, Alcohol use Smoking	Accuracy: 92.03% Sensitivity: 63.64% False Positive Rate: 6.90%
		Source of data Data collected at the Cardiovascular Diseases Division, Department of Experimental and Clinical Medicine, Faculty of Medicine, University “Magna Graecia” of Catanzaro, Italy.	Response feature No risk Risk For destabilizations within 2 week	Validation
				Leave-patient-out validation
Candelieri et al. 2009 [53]	SVM	No. of data 49 patient with CHF	Predictor features Systolic Blood Pressure (SBP), Heart Rate (HR), Respiratory Rate (RR), Body Weight (weight), Body Temperature (BT) Total Body Water (TBW). Patient condition evaluated by the cardiologist during the visit, Gender, Age, NYHA class, Alcohol use Smoking	Leave-patient-out Accuracy: 82.06% Sensitivity: 63.64% False Positive Rate: 16.90% Testing set Accuracy: 97.37% Sensitivity: 100.00% False Positive Rate: 2.78%
		Source of data Data collected at the Cardiovascular Diseases Division, Department of Experimental and Clinical Medicine, Faculty of Medicine, University “Magna Graecia” of Catanzaro, Italy.	Response feature No risk Risk For destabilizations within 2 week	Validation
				Leave-patient-out validation Testing set
Candelieri et al. 2010 [54]	SVM hyper solution framework (Genetic Algorithm)	No. of data 301 instances	Predictor features Systolic Blood Pressure, Heart Rate, Respiratory Rate, Body Weight, Body Temperature, Total Body Water), Patient health conditions, with respect to stable or decompensated status	Accuracy: 87.35% Sensitivity: 90.91% False Positive Rate: 16.21%
		Source of data Clinical study data collected through frequent follow ups	Response feature No risk Risk For destabilizations within 2 week	Validation
				Stratified 10-fold cross-validation
Guidi et al. 2014 [32]	CART Random Forests	No. of data 136 subjects 110 stable 14 rare 12 frequent	Predictor features Anamnestic data (age, gender, NYHA class) Instrumental data (weight, systolic blood pressure, diastolic blood pressure, EF, BNP, heart rate, ECG parameters (atrial fibrillation, left bundle branch block, ventricular tachycardia))	CART Accuracy: 87.60% Critical errors: 9
				Random Forests Accuracy: 85.60% Critical errors: 5
		Source of data Data collected from the Cardiology Department of the St. Maria Nuova Hospital (Florence, Italy)	Response feature Stable Rare Frequent within one year after the first visit	Validation
				A person independent ten-fold cross validation
Guidi et al. 2015 [33]	Random Forests	No. of data 250 subjects 160 none 55 rare 64 frequent	Predictor features Height and weight (Body Mass Index) Systolic and diastolic blood pressure Heart rate Oxygen saturation Ejection fraction (EF) BNP or NT-proBNP Bioelectrical impedance vector (BIVA) parameters NYHA class 12-lead EKG report (*e.g.*, presence of bundle branch block, tachycardia, atrial fibrillation, *etc.*) Etiology Comorbidity Current therapy, pharmaceutical and surgical (pacemaker or ICD ICD/CRT)	Overall accuracy: 71.90% None *vs.* all Sensitivity: 57.00% Specificity: 79.00% Rare *vs.* all Sensitivity: 65.00% Specificity: 60.00% Frequent *vs.* all Sensitivity: 59.00% Specificity: 96.00%
		Source of data Clinical study data collected through home visits and follow up	Response features Stable Rare Frequent within one year after the first visit	Validation
				10-fold cross-validation

SVM: Support Vector Machines, CHF: Congestive Heart Failure, CART: Classification and regression tree.

**Table 6:** Prediction of re-hospitalizations - review of the literature.

Authors	Method	Data	Features	Evaluation measures
Zolfaghar et al. 2013 [55]	Logistic regression Random Forests	No. of data A: 15,696 records B: 1.665.866 records (linear scale up)	Predictor features Socio demographic, vital signs, laboratory tests, discharge disposition, medical comorbidity and other cost related factors, like length of stay	Logistic regression + A Accuracy: 78.03% Precision: 33.00% Recall: 0.08% F-measure: 0.17% AUC: 59.72% Random Forests + B Accuracy: 87.12% Precision: 99.88% Recall: 40.60% F-measure: 57.37%
		Source of data National Inpatient Dataset (NIS) augment it with our patient dataset from Multicare Health System (MHS)	Response feature 30-day risk of re-admission Readmission = yes (class 1) (hospitalization within 30 days of discharge or of an earlier index of hospitalization due to CHF) Readmission = no (class 0)	Validation
				70% of the dataset train 30% of the dataset test
Vedomske et al. 2013 [56]	Random Forests	No. of data 1.000.000 patients Virginia Clinical Database Repository (CDR) Study cohort with 19.189 inpatient visits 2.749 HF diagnoses 1814 procedures	Predictor features Procedure data, diagnosis data, demographic data	With prior weighting AUC: 80% Without prior weighting AUC: 84%
		Source of data University of Virginia Clinical Database Repository (CDR) maintained by the Department of Public Health Sciences Clinical Informatics Division	Response feature Readmission within 30 days	Validation
				2/3 of the dataset used for training 1/3 of the dataset used for testing
Shah et al. 2015 [28]	SVM	No. of data 527 patients	Predictors features Phenotypic data	Area under the receiver operating characteristic curve (AUROC): 70.40% Sensitivity: 63.10% mean Specificity: 57.20% mean Precision: 63.60%
		Source of data Data collected at the outpatient clinic of the Northwestern University HFpEF Program as part of a systematic observational study of HFpEF (ClinicalTrials.gov identifier #NCT01030991)	Response feature HF hospitalization yes HF hospitalization no	Validation
				Validation set of 107 patents
Roy et al. 2015 [57]	Dynamic Hierarchical Classification	No. of data Washington State Inpatient Dataset and the Heart Failure cohort data from Multi Care Health Systems (MHS)	Predictors features Clinical. Data Socio-demographic Important data pertinent to CHF (ejection fraction, blood pressure, primary and secondary diagnosis indicating comorbidities, and APR-DRG codes for severity of illness and risk of mortality), Information about Discharges (discharge status, discharge destination, length of stay and follow-up plans) Cardiovascular and comorbidity attributes.	Accuracy: 69.20% Precision: 24.80% Recall:53.60% AUC:69.60%
		Source of data Washington State Inpatient Dataset and the Heart Failure cohort data from Multi Care Health Systems (MHS)	Response feature Readmission < 30 days Readmission > 30 days	Validation
				At each stage the best classifier was determined using a 10-fold-cross-validation procedure on training set
Koulaouzidis et al. 2016 [58]	Naïve Bayes classifier	No. of data n/a	Predictors features Blood pressure, heart rate, weight	AUC: 82%
		Source of data Kingston-upon-Hull, home telemonitoring for patients with chronic HF	Response feature High risk of HF hospitalization Low risk of HF hospitalization	Validation
				10-fold cross-validation
Kang et al. 2016 [60]	Feature selection with Bivariate analysis J48 Decision tree	No. of data 552 telemonitored HF patients	Predictors features Patient Overall status Patient living situation Severe pain experiences Frequency of activity-limiting pain Presence of skin issues Ability to dress lower body Therapy needed	AUC (c-statistic): 59% True positive rate: 65% False positive rate: 49%
		Source of data OASIS-C dataset	Response feature Likely to be hospitalized Not likely to be hospitalized	Validation
				10-fold cross-validation
Tugerman et al. 2016 [59]	Ensemble model with Boosted C5.0 tree and SVM	No. of data 20.231 inpatient admissions 4.840 CHF patients	Predictors features Comorbidities, lab values, vitals, demographics and historical	Sensitivity: 0.258 Specificity: 0.912 PPV: 0.260 NPV: 0.911 Accuracy: 0.842 F1 score: 0.259
		Source of data Veterans Health Administration (VHA) Pittsburg Hospitals	Response feature Readmission within30 days following discharge No readmission within30 days following discharge	Validation
				The data set was separated into a training set of 15,481 admissions (75%), and test (holdout/validation) set of 4840 admissions (25%).

SVM: Support Vector Machines, Sens: Sensitivity, Spec: Specificity, AUC: Area Under Curve.

**Table 7:** Prediction of mortality - review of the literature.

Authors	Method	Data	Features	Evaluation measures
Shah et al. 2015 [28]	SVM	No. of data 527 patients	Predictor features Phenotypic data	Area under the receiver operating characteristic curve (AUROC): 71.80% Sensitivity: 64.00% mean Specificity: 57.70% mean Precision: 60.90%
		Source of data Data collected at the outpatient clinic of the Northwestern University HFpEF Program as part of a systematic observational study of HFpEF (ClinicalTrials.gov identifier #NCT01030991)	Response features Death yes Death no	Validation
				Validation set of 107 patents
Fonarrow et al. 2005 [61]	CART	No. of datas 33,046 instances (derivation cohort) 32,229 instances (validation cohort)	Predictor features Demographic information, medical history, baseline clinical characteristics, initial evaluation, treatment received, procedures performed, hospital course, patient disposition	The odds ratio for mortality between patients identified as high and low risk was 12.9
		Source of data Acute Decompensated Heart Failure National Registry (ADHERE) of patients	Response features Low risk Intermediate risk 1 Intermediate risk 2 Intermediate risk 3 High risk	Validation
				Validation set of 32,229 instances
Bohacik et al. 2013 [62]	Alternating decision tree	No. of data 2032 patients	Predictor features Pulse rate, NT-proBNP level, blood sodium level, blood uric acid level, blood creatinine level, weight, height, gender, age.	Sensitivity: 37.31%, Specificity: 91.53%, PPV: 60.25%, NPV: 80.94% Accuracy: 77.66%
		Source of data Hull LifeLab - a large, epidemiologically representative, information-rich clinical database	Response features 1 year 2 years 5 years survival	Validation
				10-fold cross-validation
Panahiazar et al. 2015 [64]	Logistic regression Random Forests	No. of data 5044 HF patients	Predictor features Demographic variables, Laboratory results, Medications, 26 major chronic conditions (ICD-9 code) as comorbidities as defined by the U.S. Department of Health and Human Services.	1-year Logistic Regression AUC: 68.00% (baseline set) 81.00% (extended set) Random Forests AUC: 62.00% (baseline set) 80.00% (extended set) 2-years Logistic Regression AUC: 70.00% (baseline set) 74.00% (extended set) Random Forests AUC: 65.00% (baseline set) 72.00% (extended set) 5-years Logistic Regression AUC: 61.00% (baseline set) 73.00% (extended set) Random Forests AUC: 62.00% (baseline set) 72.00% (extended set)
		Source of data Electronic health records of the Mayo Clinic	Response features 1 year 2 years 5 years survival	Validation
				Testing set of 3484 patients
Taslimitehrani et al. 2016 [65]	CPXR(Log)	No. of data 5044 patients	Predictor features Demographics, Vitals, Lab results, Medications, 24 major chronic conditions as co-morbidities.	1-year Precision: 82.00% Recall: 78.20% Accuracy: 91.40% 2-years Precision: 78.00% Recall: 76.00% Accuracy: 83.00% 5-years Precision: 72.10% Recall: 61.50% Accuracy: 80.90%
		Source of data Electronic health records of the Mayo Clinic	Response features 1 year 2 years 5 years survival	Validation
				Testing set of 3484 patients
Austin et al. 2012 [66]	Logistic regression model (cubic smoothing splines) Boosted regression trees	No. of data EFFECT baseline (9945 HF patients) utilized 8240 EFFECT follow up (8339 HF patients) utilized 7608	Predictor features Demographic characteristics, vital signs, presenting signs and symptoms, results of laboratory investigations, and previous medical history Age, systolic blood pressure, respiratory rate, sodium, urea, history of stroke or transient ischemic attack, dementia, chronic obstructive pulmonary disease, cirrhosis of the liver, and cancer. In the CHF sample	Logistic regression model -Splines AUC: 79% R^2^: 0.203 Brier's score: 0.119 Boosted regression trees (depth four) AUC: 78% R^2^: 0.18 Brier's score: 0.107
		Source of data Enhanced Feedback for Effective Cardiac Treatment (EFFECT) Study	Response feature 30-day mortality binary variable denoting whether the patient died within 30 days of hospital admission	Validation
				EFFECT Follow-up sample was used as the validation.
Bochacik et al. 2015 [63]	Fuzzy model	No. of data n/a	Predictor features Blood Creatinine Level, Height, Blood Uric Acid Level, Age, Blood Sodium Level, Sex, Weight, NT-proBNP Level, Pulse Rate	Fuzzy model Sensitivity: 63.27% Specificity: 65.54%
		Source of data Hull LifeLab 2032 instances (HF patients)	Response feature Class attribute (patient status) classifies the patients into alive (patients being alive six and more months after the data collection) and dead (patients passing away within six months after data collection).	Validation
				10-fold cross-validation
Ramirez et al. 2015 [68]	Dichotomization thresholds Exhaustive feature selection C-SVM classifier	No. of data 597 Chronic Heart Failure patients 134 died (49 SCD victims 62 PFD victims 23 non CD victims) 463 survivors	Predictor features Δα, an index potentially related to dispersion in repolarization restitution IAA, an index reflecting the average TWA activity during a 24-h period TS, a parameter measuring the turbulence slope of HRT	SCD *vs.* the rest Sensitivity: 55% Specificity: 68% Kappa: 0.10 PFD *vs.* the rest Sensitivity: 79% Specificity: 57% Kappa: 0.14 Three-class classification SCD Sensitivity: 18% Specificity: 79% Kappa: 0.11 PFD Sensitivity: 14% Specificity: 81% Kappa: 0.11
		Source of data MUSIC (MUerte Súbita en Insuficiencia Cardiaca) study	Response feature Sudden cardiac death (SCD) Pump failure Death (PFD) Non cardiac death Survivors	Validation
				5-fold cross-validation
Subramanian et al. 2011 [67]	Ensemble Logistic regression with boosting	No. of data 963 patients	Predictor features Standard clinical variables and time-series of cytokine and cytokine receptor levels	AUC(c-statistic): 84%
		Source of data Vesnarinone Evaluation of Survival Trial (VEST)	Response feature 1 year mortality	Validation
				10-fold cross-validation

SVM: Support Vector Machines, AUC: Area Under Curve, HF: Heart Failure, PPV: Positive Predictive Values, NPV: Negative Predictive Value, CART: Classification and regression tree, CHF: Congestive Heart Failure, SCD: Sudden cardiac death, PFD: Pump failure Death, CD: Cardiac Death, *CA*: Classification Ambiguity, CIE: Cumulative Information Estimation.

**Table 8:** Advantages and disadvantages of the proposed method.

	Authors	Advantages	Disadvantages
Detection of Heart Failure	Asyali et al. 2003 [7]	Discrimination power of 9 long-term HRV measures were examined and finally only one feature SDNN is selected for the detection of HF with higher sensitivity and specificity. SDNN strong indicator for the presence of HF.	The comparison with short-term measures is limited since information regarding physical activity and sleep is not included High risk of overfitting. Neither cross-validation approach nor independent test set is used.
	Isler et al. 2007 [8]	Standard HRV measures were combined with wavelet entropy measures leading to higher discrimination power.	*k*-NN utilized by the authors lacks the property of the interpretability of induced knowledge.
	Thuraisingham 2009 [9]	Utilization of the probabilistic loss function in the CPXR(Log) algorithm. Handling of the high dimensionality and complexity of EHR data. Incorporation of information regarding comorbidities.	Information regarding the validation of the method is not provided.
	Elfadil et al. 2011 [10]	Unsupervised approach. No labeling of the dataset is needed.	Data randomly simulated are utilized for testing.
	Pecchia et al. 2011 [11]	Provides a set of rules fully understandable by cardiologists expressed as “if … then”.	The performance depends on parameter values. Methodology addressing the fact of unbalance dataset is not applied.
	Mellilo et al. 2011 [12]	Interpretability, No overfitting.	Dataset is small and unbalanced. The method is designated to cooperate with a specific classifier in the feature selection process.
	Jovic et al. 2011 [13]	HRV statistical, geometric and nonlinear measures are employed	Carefully selected collection of periods T is needed.
	Yu et al. 2012 [14]	Utilization of five category features in combination with the utilization of UCMIFS algorithm.	The value of parameter β is not determined automatically and affects the performance of the feature selector.
	Yu et al. 2012 [15]	Novel features calculated from the bispectrum are utilized.	–
	Liu et al. 2014 [16]	New nonstandard HRV measures are utilized	–
	Narin et al. 2014 [17]	Inclusion of nonlinear HRV measures and wavelet-based measures.	Unbalance dataset. Information for comorbid conditions and medication intake are not employed.
	Heinze et al. 2014 [18]	Ordinal patterns provide insight into distinctive RR interval dynamic differences. Automated relevance determination is applied in order to identify the deciding RR interval features for the discrimination between CHF and healthy subjects.	–
	Yang et al. 2010 [19]	Reliable estimation of missing values.	For the evaluation of Bayesian principal component analysis used for imputation of missing values artificial missing data are introduced to complete samples.
	Gharehchopogh et al. 2011 [20]	–	Limited number of features. Demographics, Blood Pressure and Smoking are utilized.
	Son et al. 2012 [4]	Takes into account the feature dependencies and their collective contribution.	No information regarding clinical histories, symptoms, or electrocardiogram results was exploited. The number of patients with CHF and with non-cardiogenic dyspnea was relatively small, a fact that produced variations when determining the risk factors and decision rules.
	Masetic et al. 2016 [21]	Combination of autoregressive Burg method with RF classifier.	-
	Zheng et al. 2015 [24]	The predictor features consist of cardiac reserve indexes and heart sound characteristics.	The physiological significance corresponding to the changes of indexes should be explored in depth.
Heart Failure subtypes classification	Austin et al. 2013 [26]	Boosted trees, bagged trees, and random forests do not offer an advantage over conventional logistic regression. Conventional logistic regression should remain a standard tool.	No optimization of the parameters.
	Betanzos et al. 2015 [25]	Patients belonging to “gray zone” (HFmrEF) are included in the study.	The cut-off criterion to distinguish HFpEF from HFrEF should take into consideration other information (medication, age, gender *etc*.)
	Isler 2016 [27]	HR normalization also improves the statistical significances in time-domain and non-linear HRV measures.	More patient data is needed to enhance the validity of this study.
	Authors	Advantages	Disadvantages
Severity estimation of Heart Failure	Akinyokun et al. 2009 [30]	The emotional and cognitive filters further refine the diagnosis results by taking care of the contextual elements of medical diagnosis.	Further information regarding the architecture of the neural networks are missing.
	Guidi et al. 2012 [31]	-	No justification of the selection of training (100 subjects) and testing set (36 subjects).
	Guidi et al.2014 [32]	CART provides a humanly understandable decision-making process.	Generalization of the findings is not permitted due to the small sample size.
	Guidi et al. 2015 [33]	Proposed a collaborative system for the comprehensive care of congestive heart failure.	Severity estimation of HF as mild, moderate, severe is not addressed as a three class classification problems but as a two class classification problem.
	Pecchia et al. 2011 [39]	Define mild and severe in terms of NYHA class.	No information regarding the cross-validation approach (leave-one-out, k-fold) is provided.
	Mellilo et al. 2013 [40]	Modification of the CART algorithm is proposed in order issue of imbalanced dataset to be addressed.	A larger dataset will confirm the generalization of the findings. The different extraction procedures of NN intervals. It is not clarified if the oversampling approach was applied on the construction of the tree or also to the validation.
	Yang et al.2010 [19]	Reliable estimation of missing values.	For the evaluation of Bayesian principal component analysis used for imputation of missing values artificial missing data are introduced to complete samples.
	Shahbazi et al. 2015 [41]	Combination of linear and non-linear long-term HRV measures in combination with generalized discriminant analysis.	The fact that the dataset is small and unbalanced was addressed through the leave-one-out cross-validation performance estimates. The generalization of the results is not possible due to the above mentioned fact. The sampling frequency of ECG recordings are not equal. The procedures of extracting NN intervals are not the same.
	Sideris et al. 2015 [45]	A novel data-driven framework to extract predictive features from disease and symptom diagnostic codes is proposed. Number of cluster-based features is automatically determined through a greedy optimization methodology.	Further information regarding the definition of the six daily threshold-based outcome variables is needed (why only heart rate and systolic blood pressure is included, does the ranges of these measures are differentiated depending on the patient)
Prediction of adverse events Destabilization	Candelieri et al. 2008 [52]	Presented a decision tree which was evaluated in terms of predictive performance (accuracy and sensitivity) through a suitable validation technique and it was checked by clinical experts in terms of plausibility.	Low sensitivity.
	Candelieri et al. 2009 [53]	–	Only 1 of the 4 patients belonging to testing set but not to training set, have presented a decompensation.
	Candelieri et al. 2010 [54]	SVM hyper solution framework performing, at the same time, Model Selection, Multiple Kernel Learning and Ensemble Learning with the aim to identify the best hyper-classifier is proposed.	–
	Guidi et al.2014 [32]	CART provides a humanly understandable decision-making process.	Generalization of the findings is not permitted due to the small sample size.
	Guidi et al. 2015 [33]	Proposed a collaborative system for the comprehensive care of congestive heart failure.	–
	Authors	Advantages	Disadvantages
Prediction of adverse events Re-hospitalizations	Zolfaghar et al. 2013 [55]	A big data solution for predicting the 30-day risk of readmission for the CHF patients is proposed.	–
	Vedomske et al. 2013 [56]	Incorporation of billing information in the prediction of re-hospitalizations.	Data from a single hospital are employed. Visits which contained no data for readmissions were excluded.
	Shah et al. 2015 [28]	Relationship between the pheno-groups and adverse outcomes.	Further demonstration of generalizability is needed.
	Roy et al. 2015 [57]	Hierarchical classification technique for risk of readmission, dividing the prediction problem in several layers, is proposed. Algorithmic layering capability is trained and tested over two real world datasets and is currently integrated into the clinical decision support.	–
	Koulaouzidis et al. 2016 [58]	Telemonitoring data are employed.	Small number of predictor features. Utilization of different classifiers. No information regarding the sample size.
	Kang et al. 2016 [60]	It provides a preliminary understanding of the characteristics of telehomecare patients that were associated with re-hospitalization. It provides a visual depiction of the associations among risk factors, allowing a more complete exploration of the profile of patients at high risk for re-hospitalization among all patients who used telehomecare.	Input variables does not include (bio)markers or characteristics of medication noncompliance that may affect re-hospitalization.
	Tugerman et al. 2016 [59]	A mixed-ensemble model for predicting hospital readmission is proposed. An optimization approach, which takes into account the degree of correlation between the models, the distance of the minority instances to the decision boundaries of the SVM, the penalty for misclassification errors for patients who were actually readmitted (positive readmission instances), and generalization power is proposed.	The dataset is highly imbalanced.
Prediction of adverse events Mortality	Shah et al. 2015 [28]	Relationship between the pheno-groups and adverse outcomes.	Further demonstration of generalizability is needed.
	Fonarrow et al. 2005 [61]	5 levels of risk are estimated.	Each patient's actual risk may be influenced by many factors not measured or considered in this model.
	Bohacik et al. 2013 [62]	Alternating decision trees allows the estimation of the contribution of each decision node in isolation.	Low sensitivity.
	Panahiazar et al. 2015 [64]	Hazard Ratio (HR) is calculated based on real world EHR data.	-
	Taslimitehrani et al. 2016 [65]	CPXR(Log) is used allowing effectively building of highly accurate prediction models on datasets with diverse predictor– response relationships	The selection of the parameters values affecting CPXR(Log) is not justified.
	Austin et al. 2012 [66]	-	Utilization of other classifiers is not employed. Regression models did not include shrinkage or penalized estimation methods
	Bochacik et al. 2015 [63]	Interpretability was evaluated using quantitative measures. An algorithmic model using computations of ambiguity and utilizing notions of fuzzy logic is proposed.	-
	Ramirez et al. 2015 [68]	Different etiologies of mortality are predicted.	The utilization of fully automated ECG measurements may induce imprecision. The number of SCD and PFD victims was relatively low in comparison with survivors.
	Subramanian et al. 2011 [67]	A multivariate logistic regression model using baseline and serial measurements of cytokine and cytokine receptors levels up to 24 weeks predicts 1-year mortality.	-

## Footnotes

1 http://physionet.org/physiobank/database/mitdb/.

2 http://archive.ics.uci.edu/ml/datasets/Heart+Disease.

3 http://www.hcup-us.ahrq.gov/sidoverview.jsp.

4 https://www.multicare.org/.

5 http://arxiv.org/abs/1602.03686.
